# Comprehensive causes of death in uveal melanoma: mortality in 1530 consecutively diagnosed patients followed until death

**DOI:** 10.1093/jncics/pkad097

**Published:** 2023-11-16

**Authors:** Gustav Stålhammar

**Affiliations:** Department of Clinical Neuroscience, Division of Eye and Vision, Karolinska Institutet, Stockholm, Sweden; Ocular Oncology Service and St. Erik Ophthalmic Pathology Laboratory, St. Erik Eye Hospital, Stockholm, Sweden

## Abstract

**Background:**

Uveal melanoma has a high propensity for metastatic spread. Yet, the comprehensive causes of death in a large consecutive cohort followed from diagnosis to death remain unknown.

**Methods:**

All Swedish patients diagnosed with melanoma involving the iris, choroid, and/or ciliary body after January 1, 1960, were assessed for this study. Sequential inclusion was halted upon encountering the first surviving patient during data collection. Causes of death were collected from the National Cause of Death Registry and audited by analysis of up to 15 causative diagnoses.

**Results:**

A total of 1530 patients were included, each histopathologically verified with primary uveal melanoma. Mortality from metastatic uveal melanoma was 31% at 5 years, 40% at 10 years, 45% at 20 years, 47% at 30 years, and 48% between 40 and 60 years post-diagnosis. Notably, the longest period between diagnosis and metastatic fatality was 49.6 years. Additionally, 186 other causes of death were recorded, with cardiovascular diseases constituting 26%, other cancers 10%, stroke 6%, dementias 2%, and lower respiratory infections 2% of total mortalities. Mortality from colorectal, lung, prostate, and stomach carcinomas over 60 years were 1.4%, 1.4%, 1.2%, and 0.9%, with metastatic uveal melanoma being the leading cumulative and annual cause of death for the initial 41 and 5 years post-diagnosis, respectively.

**Conclusions:**

In this large consecutive cohort, half of the included patients ultimately succumbed to metastatic uveal melanoma, with deaths occurring up to 50 years after diagnosis. One-quarter and one-tenth of patients died from cardiovascular diseases and other cancers, respectively.

Uveal melanoma is the most prevalent intraocular cancer in adults, characterized by a high risk of metastatic spread. Despite the recent introduction of treatment strategies that have improved median overall survival for patients with metastatic disease, the prognosis in this disease remains poor (1).

Previous estimations of the proportion of patients who die from uveal melanoma vary considerably. Some publications report mortality rates in the range of 20% to 25%, whereas others who have followed similar patients over similar periods of time have reported rates twice as high (2-10). These discrepancies may be partially attributable to differences in methods for data collection, for classification of causes of death, and for survival calculations. Actuarial methods, such as life tables and Kaplan-Meier estimates, are excellent for evaluating all-cause mortality but tend to overestimate disease-specific mortality in the presence of competing risks, including death from other causes (11-13). In a 2003 Finnish study of patients treated with primary enucleation, mortality from metastatic uveal melanoma (UM mortality) was 52% at 35 years (11). In a more recent study of all Swedish patients diagnosed between 1980 and 2017, with a smaller mean tumor size, UM mortality was 36% at 30 years (14). However, considering the tremendously long follow-up required to track a large consecutive cohort in which all patients have deceased, there are no complete reports on the causes of death in uveal melanoma. Even with very long follow-up, a substantial proportion of survivors have remained, and the causes of death for the patients surviving longest after their uveal melanoma diagnosis are poorly understood.

Therefore, we followed a cohort of 1530 consecutive patients from diagnosis to death. These patients represent all patients diagnosed with uveal melanoma in Sweden between 1960 and 1979. We find that 48% of patients died from metastatic uveal melanoma; that metastatic uveal melanoma was the leading cumulative and annual cause of death during the first 41 and 5 years after uveal melanoma diagnosis, respectively; that non-melanoma-related deaths were distributed over 186 other causes; and that 26%, 10%, and 6% of uveal melanoma patients died from cardiovascular diseases, other cancers, and stroke.

## Methods

### Study population

All patients diagnosed with uveal melanoma (involving the iris, choroid, and/or ciliary body) at St. Erik Eye Hospital, Stockholm, Sweden, since the beginning of our records on January 1, 1960, were considered for the study. Throughout the period, St. Erik Eye Hospital was the only institution in Sweden to diagnose uveal melanoma, ensuring full capture of all such patients in the country. A sequential inclusion procedure was used to follow all patients from diagnosis to death; patients diagnosed between January 1, 1960, and August 3, 1979, were included in the analysis. Some patients diagnosed after August 3, 1979, were still alive at the time of data collection, so in order to include only consecutive patients who had died, patients diagnosed after this date were not considered. Diagnostic procedures and trends in patient age, tumor size, incidence rates, and survival since 1960 have been described previously (15). The following data were collected: patient sex; patient age at diagnosis; patient age at death; diagnosis date; death date; underlying cause of death, causative diagnoses; emigration status; and what version of the International Classification of Diseases (ICD) had been used. All patients had been treated with primary enucleation, as plaque brachytherapy was introduced in late 1979. The study adhered to the tenets of the Declaration of Helsinki, and approval was obtained from the Swedish Ethical Review Authority (reference 2022 06725 02). Informed consent was waived because this was a retrospective chart review that did not include living patients and did not include analyses of biological tissues. The STROBE reporting guidelines for cohort studies were used.

### Determining cause of death from death certificates

For each patient, the cause of death certificate specified up to 15 causative diagnoses that led to an underlying cause of death. For example, if a patient with extensive metastatic disease in the brain, lungs, liver, and myocardium (causative diagnoses) developed organ failure and died from circulatory insufficiency, the underlying cause of death was classified as metastatic uveal melanoma and not cardiovascular disease. For a detailed account on the method for establishing the cause of death for each patient, please see the Supplementary Material (available online).

### Statistical analysis

For tests of continuous variables that did not deviate statistically significantly from normal distribution (Shapiro–Wilk test *P *>* *.05), Student’s *t* tests were used. For nonparametrical data, Mann–Whitney *U* tests were used. Cumulative and annual incidence function point estimates and 95% confidence intervals (CIs) were calculated from competing risks data, and the equality of survival distributions was tested with Gray’s test. Additionally, relative incidence was calculated by dividing the incidence of death from metastatic uveal melanoma by the incidence of death from other causes. Kaplan-Meier curves were drawn for each major cause of death but were expected to provide exaggerated point estimates in late follow-up periods (eg, >20 years after diagnosis) (11). Relative survival was defined as the ratio of the observed Kaplan-Meier overall survival among the 1530 included patients to the Kaplan-Meier overall survival of an equally large group of individuals of the same sex and age from the general population. The latter data were collected from Statistics Sweden life expectancy tables (16). The 95% confidence intervals of the relative survival rate were calculated as:


$$CI=\hat{p}\mp 1.96\sqrt{\frac{\hat{p}(1-\hat{p})}{n}}$$

where $\hat{p}$ is the point estimate of the relative survival rate, and *n* is the number of remaining patients at the corresponding point in time. *P* values less than .05 were considered statistically significant, all *P* values being two-sided. Competing risk incidences were calculated with the cmprsk package for R (R Foundation for Statistical Analysis, Vienna, Austria) and Kaplan-Meier curves with the survival and survminer packages (17). All other statistical analyses were performed using IBM SPSS Statistics, version 27 (Armonk, NY, USA) or GraphPad Prism, version 9.3.0 (San Diego, CA, USA).

## Results

### Descriptive statistics

**Figure 1.**
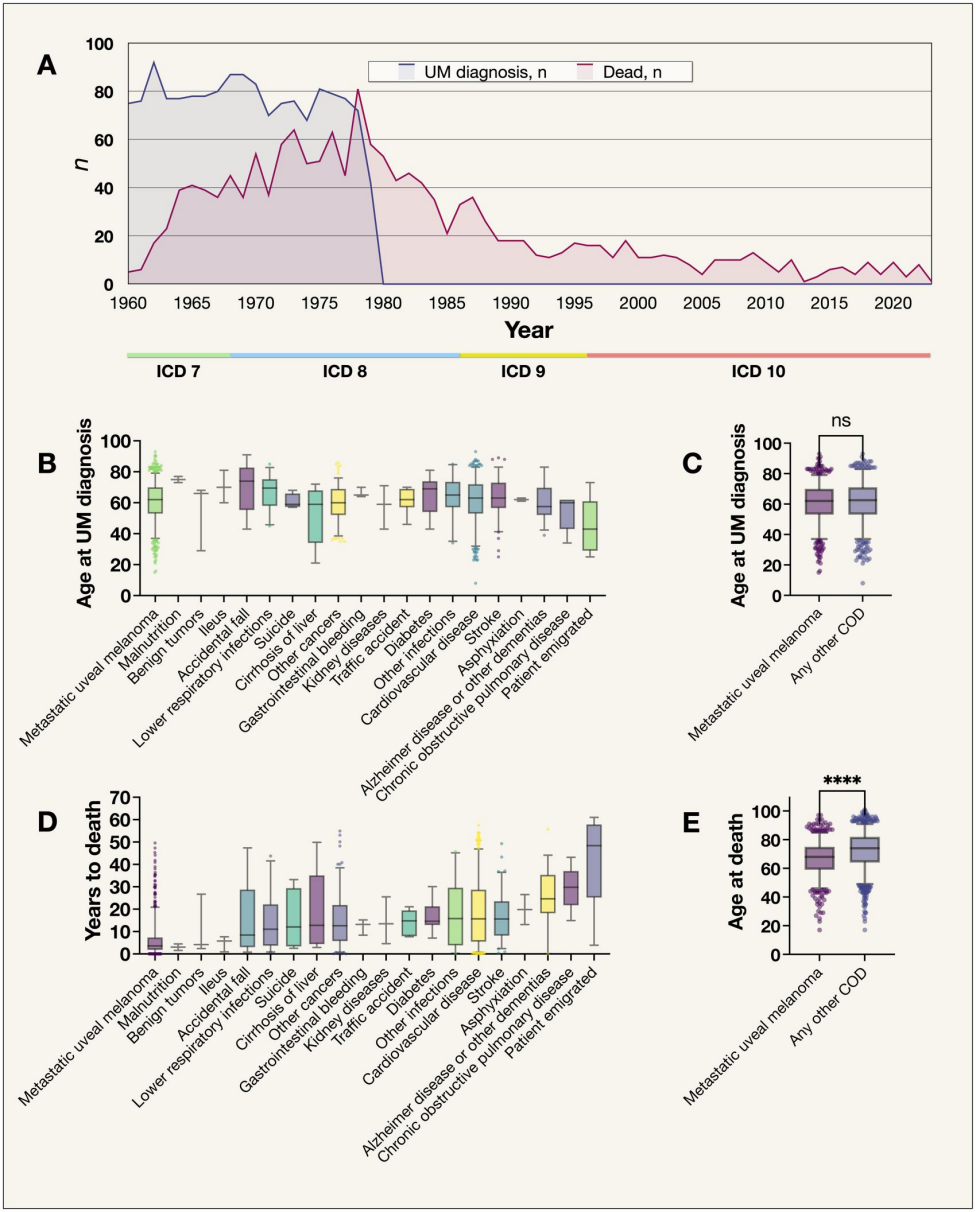
Age and follow-up characteristics of the included patients. **A**) The 1530 included patients constitute all Swedish patients diagnosed between January 1, 1960, and August 3, 1979. The last one of these 1530 patients died in early 2023. Each patient’s underlying COD was classified according to the International Classification of Diseases (ICD), 7th version (ICD 7) between 1960 and 1965, ICD 8 between 1966 and 1986, ICD 9 between 1987 and 1996, and ICD 10 from 1997. **B**) Patient age at the time of primary uveal melanoma diagnosis, by cause of death (COD) group. Five patients emigrated during the period, and, for these patients, vital status and COD were unavailable. **C**) Patient age at the time of UM diagnosis for patients that eventually deceased from metastatic UM versus any other COD. The difference was not statistically significant on a .05 level (Mann-Whitney *U P *=* *.13). **D**) Observation time from uveal melanoma diagnosis to death, by COD group. **E**) Patients who died from metastatic UM were statistically significantly younger at the time of death than patients who died from any other cause. ****P <* *.0001. COD = cause of death; ICD = International Classification of Diseases by the World Health Organization; ns = statistically nonsignificant; UM = uveal melanoma. Centerlines in B through E denote median values, whereas the boxes contain the 25th to 75th percentiles of the data sets. Whiskers indicate 5th to 95th percentile.

The 1530 included patients constitute all Swedish patients diagnosed between January 1, 1960, and August 3, 1979. All patients had been treated with primary enucleation, as plaque brachytherapy was introduced in late 1979, and all primary uveal melanoma diagnoses were confirmed histopathologically. Seven hundred twenty-four patients (47%) were female (Table 1). The mean largest basal tumor diameter and thickness was 12.1 and 7.9 mm, respectively. Five patients (<1%) emigrated, and the cause of death was not available for these patients. The highest frequency of deaths occurred before 1985, with relatively few patients surviving into the 2000s (Figure 1, A). The number of patients diagnosed and deceased each year 1960–2023 is provided in Supplementary Table 1 (available online). Their mean age at diagnosis was 61 years (standard deviation [SD] = 13; Figure 1, B). There was no statistically significant difference in the patient age at the time of uveal melanoma diagnosis between those who died from metastatic uveal melanoma and any other cause (62.5 years vs 62.5 years, respectively; Mann-Whitney *U P *=* *.13; Figure 1, C). The median number of years between uveal melanoma diagnosis and death was longest for those who died from chronic obstructive pulmonary disease and Alzheimer disease or other dementias, whereas it was shortest for those who died of metastatic uveal melanoma (n =* *736) and malnutrition (n =* *2; Figure 1, D). Patients who died from metastatic uveal melanoma were statistically significantly younger at the time of death (68.0 years) than those who died from other causes (74.0 years, *P *<* *.001; Figure 1, E).

**Table 1.** Clinicopathological characteristics of included patients^a^

Sex, No. (%)	
Female	724 (47)
Male	806 (53)
Age at diagnosis, mean years (SD)	61 (13)
Age at death, mean years (SD)	73 (13)
Tumor LBD, mean mm (SD)	12.1 (4.1)
Tumor thickness, mean mm (SD)	7.9 (3.0)
Median follow-up, years (IQR, min–max)	
All patients (*n *=* *1530)	6.8 (15.1, 0.0–61.0)
Dead from MUM (*n *=* *736)	3.6 (5.6, 0.0–49.6)
Dead from other causes (*n *=* *794)	15.2 (20.1, 0.0–61.0)

a IQR = interquartile range; LBD = largest basal diameter; MUM = metastatic uveal melanoma; SD = standard deviation.

### Audited cause of death

When accounting for overlap of disease terms between ICD versions, there were 186 underlying causes of death in the data (Supplementary Table 2, available online). The audited cause of death was metastatic uveal melanoma in 736 patients (48%). This was based on an underlying cause of death registered as cutaneous melanoma (n =* *256), malignant neoplasm of the eye (n =* *223), malignant neoplasm of the choroid (n =* *186), secondary neoplasm with specified or unspecified site but with melanoma as a causative diagnosis (n =* *48), or malignant neoplasm without specification of site but with melanoma as a causative diagnosis (n =* *23).

Three hundred ninety-eight patients (26%) died from cardiovascular diseases. Of these 398, 137 (9% of all 1530 patients, 34% of cardiovascular diseases) died from chronic ischemic heart disease, and 134 (9% of all 1530 patients, 34% of cardiovascular diseases) from acute myocardial infarction.

One hundred fifty-five (10%) patients died from other cancers, of which colorectal carcinoma (n =* *22, 1%), lung cancer (n =* *21, 1%) prostate cancer (n =* *19, 1%), and stomach cancer (n =* *14, 1%) were the most common. Eighty-five patients (6%) died from stroke, 36 patients (2%) from Alzheimer disease or other dementias, and 34 from lower respiratory infections (eg, pneumonia; Table 2).

**Table 2.** Underlying cause of death for all 1530 patients, simplified

Cause of death	No.	%	Relative incidence
Metastatic uveal melanoma	736	48.1	1
Cardiovascular disease	398	26.0	0.54
Other cancers	155	10.1	0.21
Stroke	85	5.6	0.12
Alzheimer disease or other dementias	36	2.4	0.05
Lower respiratory infections (eg, pneumonia)	34	2.2	0.05
Other infections	21	1.4	0.03
Accidental fall	12	0.8	0.02
Complications of diabetes	11	0.7	0.01
Traffic accident	7	0.5	0.01
Chronic obstructive pulmonary disease	5	0.3	0.01
Cirrhosis of liver	5	0.3	0.01
Patient emigrated (ie, cause of death not available)	5	0.3	0.01
Suicide	4	0.3	0.01
Benign tumors	3	0.2	0.00
Gastrointestinal bleeding	3	0.2	0.00
Ileus	3	0.2	0.00
Kidney diseases	3	0.2	0.00
Asphyxiation (eg, foreign body in larynx)	2	0.1	0.00
Malnutrition	2	0.1	0.00
*Sum*	*1530*	*100.0*	

### Cumulative incidence of death

**Figure 2.**
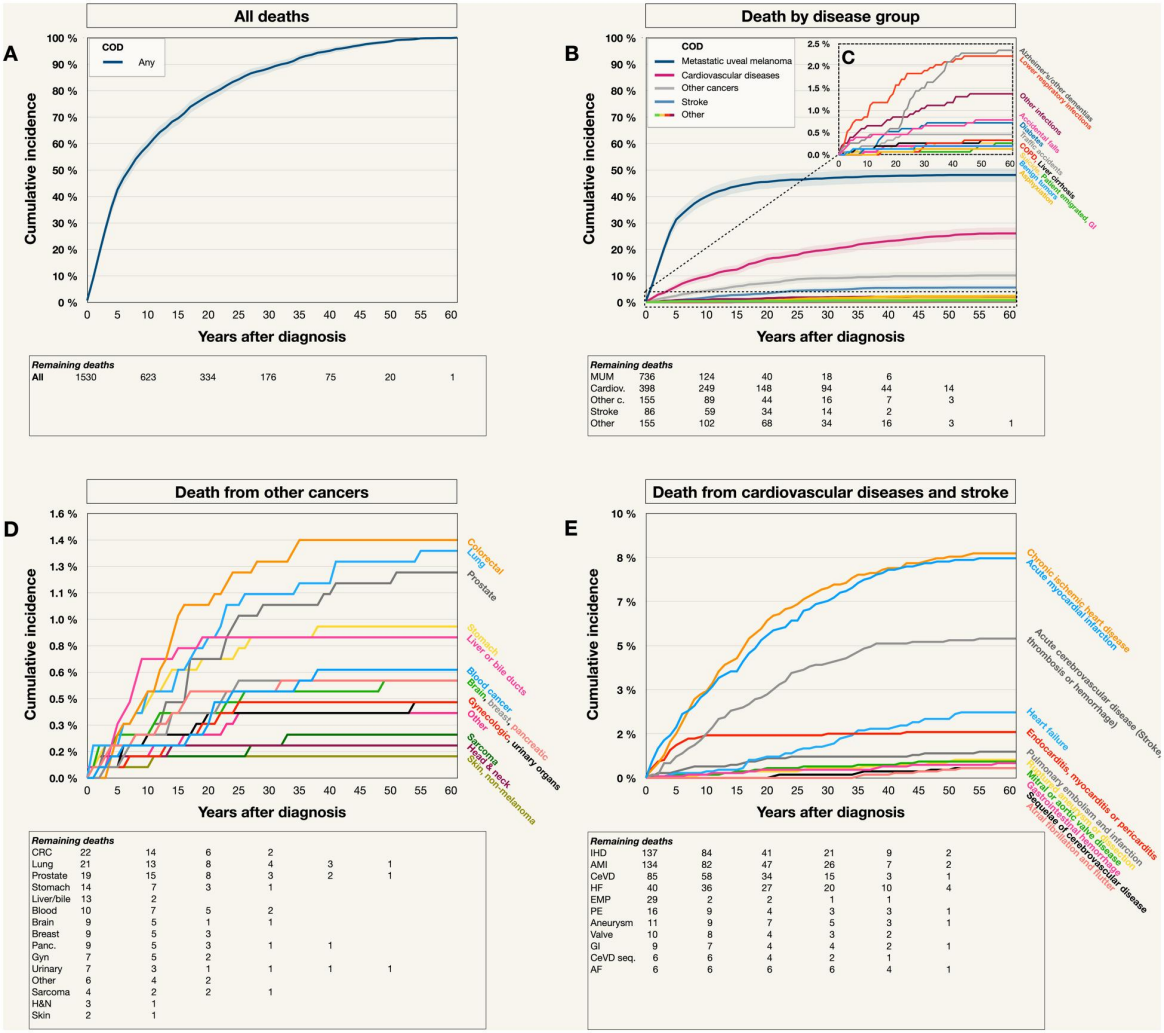
Cumulative incidences. **A**) Death from any cause. **B**) Death by disease group (metastatic uveal melanoma, cardiovascular disease, other cancers, stroke, or other causes). **C**) Insert, illustrating curves for deaths with a cumulative incidence of 0 to 2.5%. **D**) Death from cancers other than metastatic uveal melanoma. **E**) Death from cardiovascular diseases and stroke. Colored fields in A and B represent 95% confidence intervals. AF = atrial fibrillation and flutter; AMI = acute myocardial infarction; Cardiov. = cardiovascular diseases; CeVD = acute cerebrovascular disease; CeVD seq. = sequelae of cerebrovascular disease; COD = cause of death; COPD = chronic obstructive pulmonary disease; CRC = colorectal carcinoma; EMP = endocarditis, myocarditis, or pericarditis; GI = gastrointestinal hemorrhage; Gyn. = gynecologic cancer; H&N = head and neck carcinoma; HF = heart failure; IHD = ischemic heart disease; MUM = metastatic uveal melanoma; Other c = other cancer; Panc. = pancreatic carcinoma; PE = pulmonary embolism’ Valve, mitral or aortic valve disease. Note that the scale on the *y*-axis varies.

All-cause mortality was 43% at 5 years, 59% at 10 years, 78% at 20 years, and 100% at 61 years (Figure 2, A).

UM mortality was 31% at 5 years, 40% at 10 years, 44% at 15 years, 45% at 20 years, 46% at 25 years, 47% at 30 years, 47% at 35 years, and 48% at 40 to 60 years after diagnosis. The longest period between uveal melanoma diagnosis and metastatic death was observed for a woman who was diagnosed in 1969 and died from metastatic disease 49.6 years later, in 2019. No death from metastatic uveal melanoma was observed between 49.6 and 61 years post-diagnosis. Metastatic uveal melanoma was the leading cumulative cause of death for the first 41 years.

The incidence curve for death from cardiovascular diseases increased gradually over the entire 60-year observation period, with a cumulative incidence of 6% at 5 years, 10% at 10 years, 12% at 15 years, 16% at 20 years, 18% at 25 years, 20% at 30 years, 22% at 35 years, 23% at 40 years, 24% at 45 years, 25% at 50 years, and 26% at 55 and 60 years.

Mortality from other cancers rose for the first 25 years after uveal melanoma diagnosis, after which the curve flattened, with a cumulative incidence of 2% at 5 years, 4% at 10 years, 6% at 15 years, 7% at 20 years, 9% at 25 to 35 years, and 10% at 40 to 60 years. Mortality from stroke rose for the first 25 years after uveal melanoma diagnosis, with a cumulative incidence of 1% at 5 years, 2% at 10 years, 3% at 15 and 20 years, 4% at 25 years, 5% at 30 to 45 years, and 6% at 50 to 60 years (Figure 2, B). Among less common deaths (cumulative incidence <2.5%), Alzheimer disease and other dementias, lower respiratory infections, other infections, accidental falls, and complications of diabetes were the most common. Death from Alzheimer disease and other dementias had an S-shaped incidence curve, being relatively uncommon during the first 20 years after uveal melanoma diagnosis, and then increasing rapidly between 20 and 40 years, after which the incidence plateaued at 2.4% (Figure 2, C).

Other cancer deaths included colorectal, lung, prostate, stomach, liver or bile duct, breast, pancreatic, gynecologic, urinary tract, head and neck, and non-melanoma skin carcinomas, blood cancers, and sarcoma. The 60-year mortalities from colorectal, lung, prostate, and stomach carcinoma were 1.4%, 1.4%, 1.2%, and 0.9%, respectively (Figure 2, D).

Death from chronic ischemic heart disease virtually paralleled the cumulative incidence curve for death from acute myocardial infarction, reaching 8% at 34 and 37 years after uveal melanoma diagnosis, respectively. Mortality from endocarditis, myocarditis, or pericarditis reached 2% at 9 years after uveal melanoma diagnosis, after which it plateaued and was surpassed by the cumulative incidence curve of death from heart failure, which reached more than 2% 35 years after uveal melanoma diagnosis (Figure 2, E).

### Causes of death 20 to 60 years after uveal melanoma diagnosis

In the period more than 20 years after uveal melanoma diagnosis, UM mortality increased from 45% to 48%; from cardiovascular diseases from 16% to 26%; from other cancers from 7% to 10%; and from stroke from 3% to 6%. In terms of annual incidence, death from cardiovascular diseases were more common than death from metastatic uveal melanoma. Mortality from other cancers essentially matched the UM mortality. More than 50 years from uveal melanoma diagnosis, the annual mortality in the remaining population was low for most conditions, with accidental falls being among the top 3 causes of death (Supplementary Figure 1, available online).

As expected, Kaplan-Meier analyses appeared to inflate long-term mortality estimates. When observed 50 years post-diagnosis, the melanoma-specific survival proportion was calculated to be 33% (95% CI = 28% to 39%). This translates to a mortality from uveal melanoma being overstated by 19 percentage points (derived from 1 minus the melanoma-specific survival: 67%, 95% CI = 61% to 72%) when compared to the 48% UM mortality (95% CI = 46% to 51%) as determined by competing risk analysis. The inflationary effect became notably perceptible in the context of cardiovascular diseases, which were characterized by a relatively large number of late events (Supplementary Figure 2, available online).

### Relative survival

**Figure 3.**
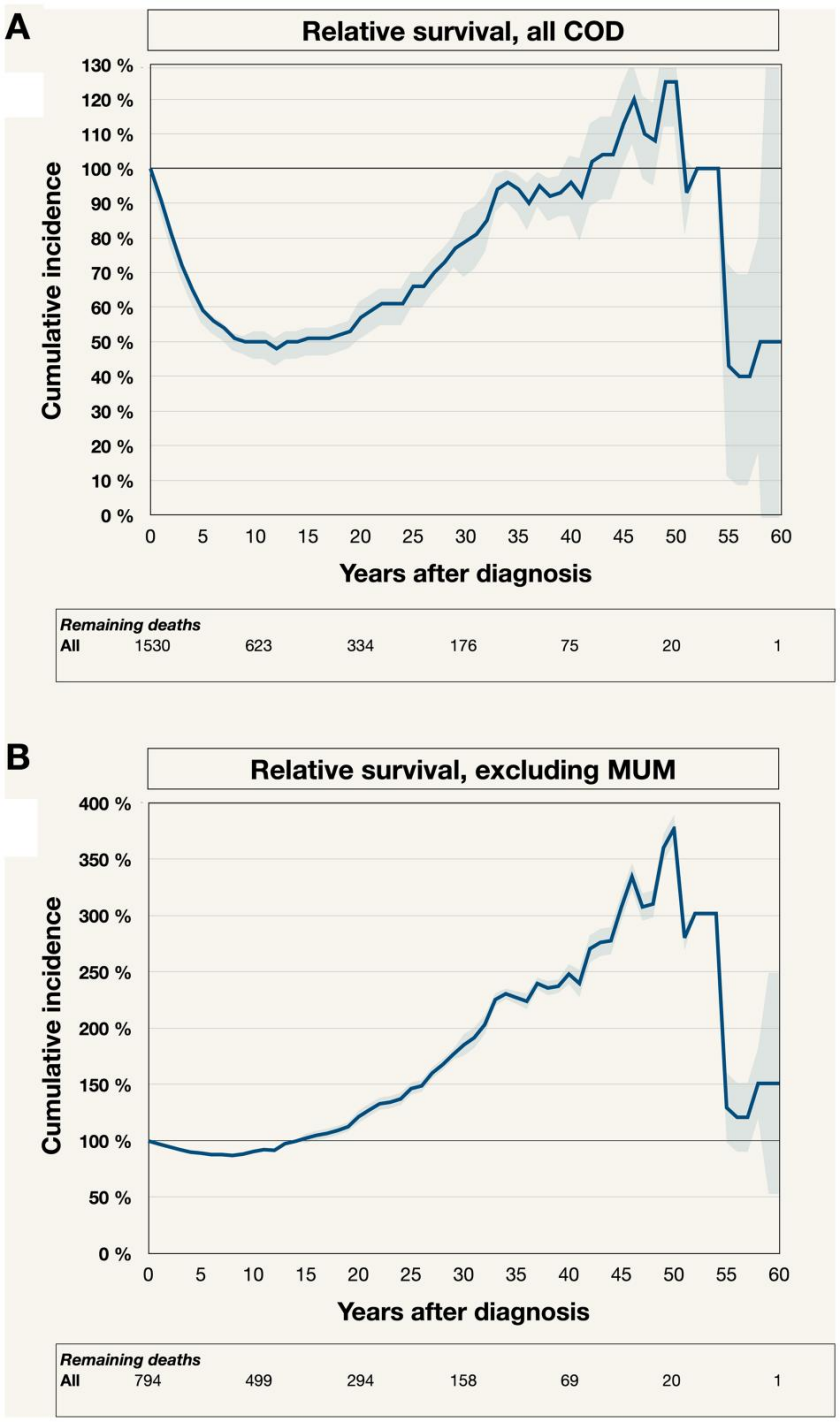
Relative survival analysis. **A**) Kaplan-Meier overall survival for all 1530 patients, accounting for any COD, divided by the expected overall survival for a sample of individuals with identical sex and age from the general population. **B**) Relative survival for all 1530 patients, excluding deaths from metastatic uveal melanoma (MUM). Colored fields in A, B, and C indicate 95% confidence intervals. COD = cause of death; MUM = metastatic uveal melanoma. Note that the scale on the *y*-axis varies.

The 1530 patients diagnosed with uveal melanoma had a rapidly declining relative survival rate in the first decade after diagnosis, reaching 48% during the 12th year after diagnosis. However, in the second decade, relative survival stabilized, followed by an upward trajectory. By the fourth decade, relative survival rates ranged from 81% to 93%, ultimately reaching a peak of 102% to 125% between 42 and 50 years post-diagnosis (Figure 3, A).

When deaths from metastatic uveal melanoma were excluded, higher relative survival rates were observed, as expected. These rates reached their lowest point at 87% during the eighth year after diagnosis, subsequently exceeding 100% after 14 years post-diagnosis. It is essential to note that, upon censoring deaths due to metastatic uveal melanoma, patients affected by this condition were no longer at risk of succumbing to other causes (Figure 3, B).

### Annual and relative incidence of death

**Figure 4.**
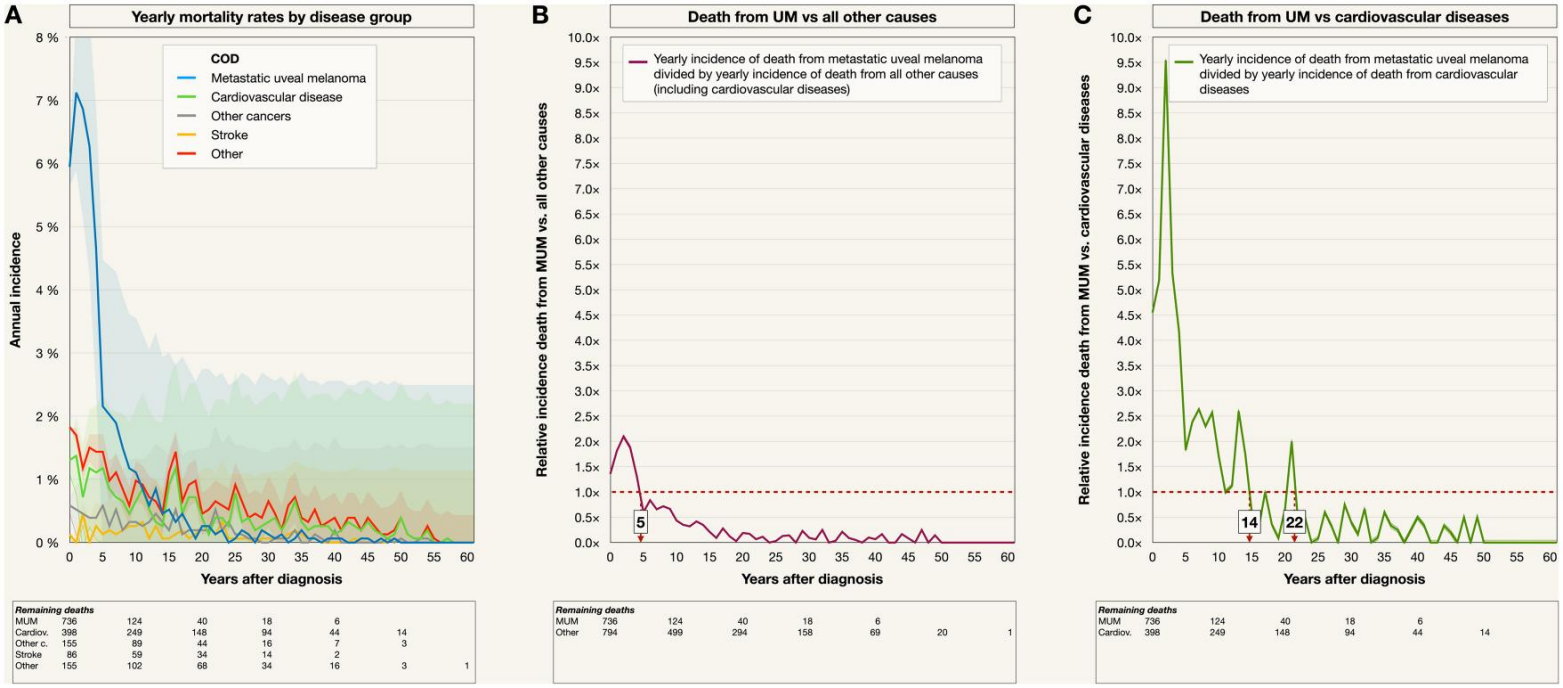
Annual mortality. **A**) Death by disease group (metastatic uveal melanoma, cardiovascular disease, other cancers, stroke, or other causes). **B**) Relative annual mortality from metastatic uveal melanoma (UM mortality) to mortality from any other cause. UM was the leading cause of death during the first 5 years after uveal melanoma diagnosis. **C**) Relative annual UM mortality to mortality from cardiovascular diseases. UM was a more common cause of death (COD) than cardiovascular diseases during the first 22 years after uveal melanoma diagnosis. Colored fields in A, B, and C indicate 95% confidence intervals. Cardiov. = cardiovascular diseases; Other c = other cancer.

A closer examination of annual mortality rates revealed that UM mortality peaked at 7% during the second and third year after diagnosis, after which it declined to less than 1% in the period more than 10 years after diagnosis (Figure 4, A). Metastatic uveal melanoma was the leading annual cause of death over all other causes combined for the first 5 years after diagnosis. The rate of death from metastatic uveal melanoma to death from any other cause was 1.9 to 2.9× during the first 5 years after diagnosis, with the peak reaching 2.9× during the fourth year (Figure 4, B). Relative to the annual rate of death from cardiovascular diseases, metastatic uveal melanoma was the leading cause of the for the first 14 years, after which it was superseded by cardiovascular diseases for the remainder of the period (except for the 22nd year). The rate of death from metastatic uveal melanoma to death from cardiovascular diseases was 4.2 to 9.5× during the first 5 years after diagnosis, with the peak reaching 9.5× during the thirrd year (Figure 4, C).

### Death causes by patient sex and age at diagnosis

**Figure 5.**
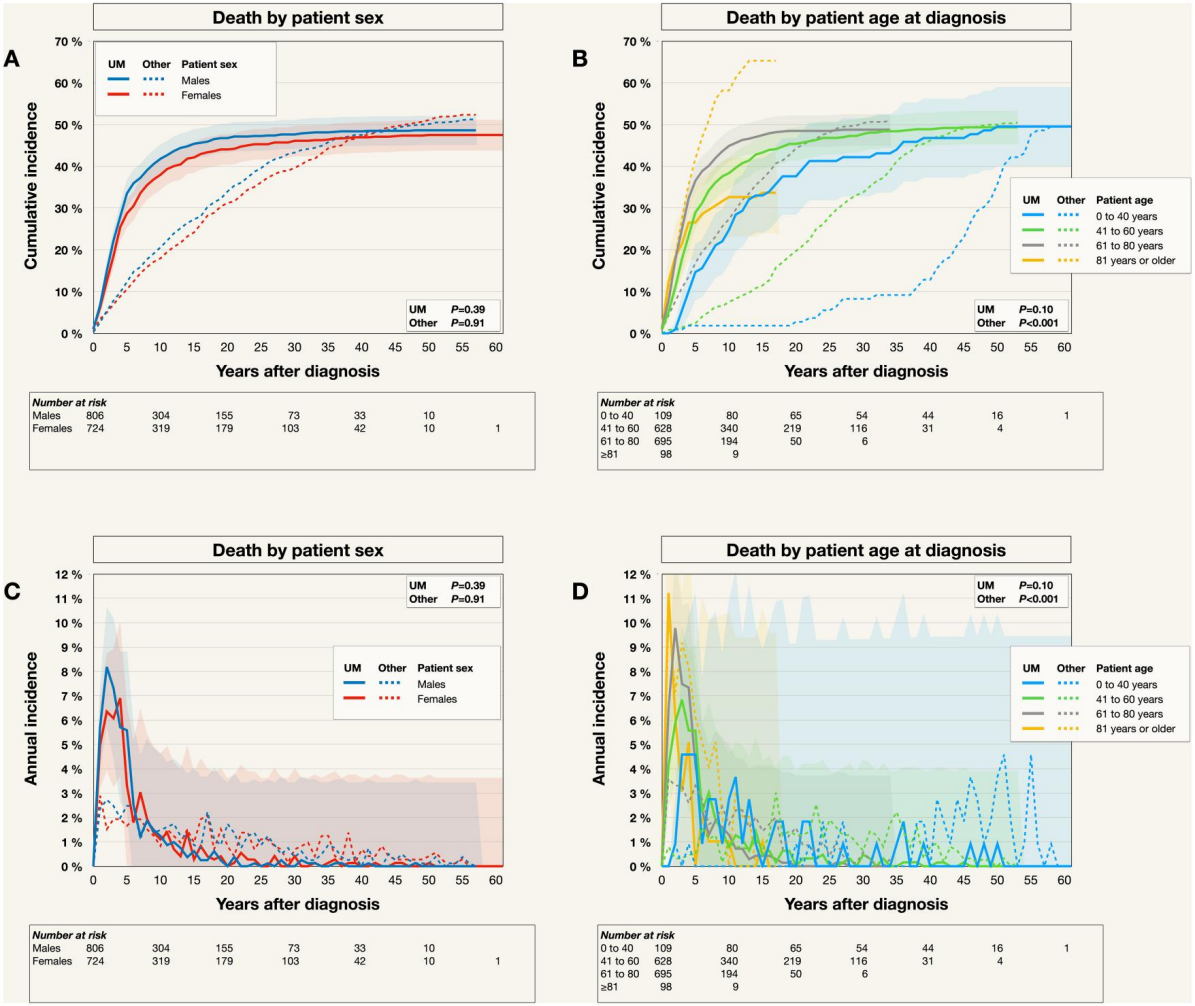
Cumulative mortality from metastatic uveal melanoma (UM mortality) and other causes by patient sex and age. **A**) Cumulative UM mortality (**solid lines**) and mortality from any other cause (**dashed lines**) in males versus females. **B**) Cumulative UM mortality (**solid lines**) and mortality from any other cause (**dashed lines**) in patients who were 40 years of age or younger at the time of UM diagnosis, 41 to 60 years, 61 to 80 years, and 81 years or older. The youngest group had lower UM mortality during the first 2 decades after diagnosis, but long-term survivors reach a similar incidence as older patients. The oldest group had markedly lower UM mortality at 15 years, associated with a much higher mortality from other causes. Colored fields in A and B indicate 95% confidence intervals.

There were no statistically significant differences in UM mortality between male and female patients (Gray’s *P *=* *.39), or in mortality from any other cause (*P *=* *.91). Metastatic uveal melanoma was the leading cumulative cause of death for the first 43 and 39 years after diagnosis for male and female patients, respectively (Figure 5, A).

Similarly, there were no statistically significant differences in UM mortality between patients who were 40 years of age or younger, 41 to 60 years, 61 to 80 years, or 81 years or older at the time of diagnosis (*P *=* *.10). As expected, mortality from other causes differed statistically significantly: 42% of patients aged at least 81 years; 17% of patients aged 61 to 80 years; 2% of patients aged 41 to 60 years; and 2% of patients aged 40 years of age or younger died from other causes within 5 years from uveal melanoma diagnosis (*P *<* *.001, Figure 5, B).

In terms of annual incidence, male patients peaked with a UM mortality of 8% during the third year after diagnosis, whereas female patients peaked with 7% during the fifth year (Figure 5, C). Patients who were at least 81 years at the time of diagnosis had a peak annual incidence rate of death from metastatic uveal melanoma at 11% during the second year, whereas patients who were 40 years of age or younger peaked at 5% during the fourth through the sixth year (Figure 5, D).

**Figure 6.**
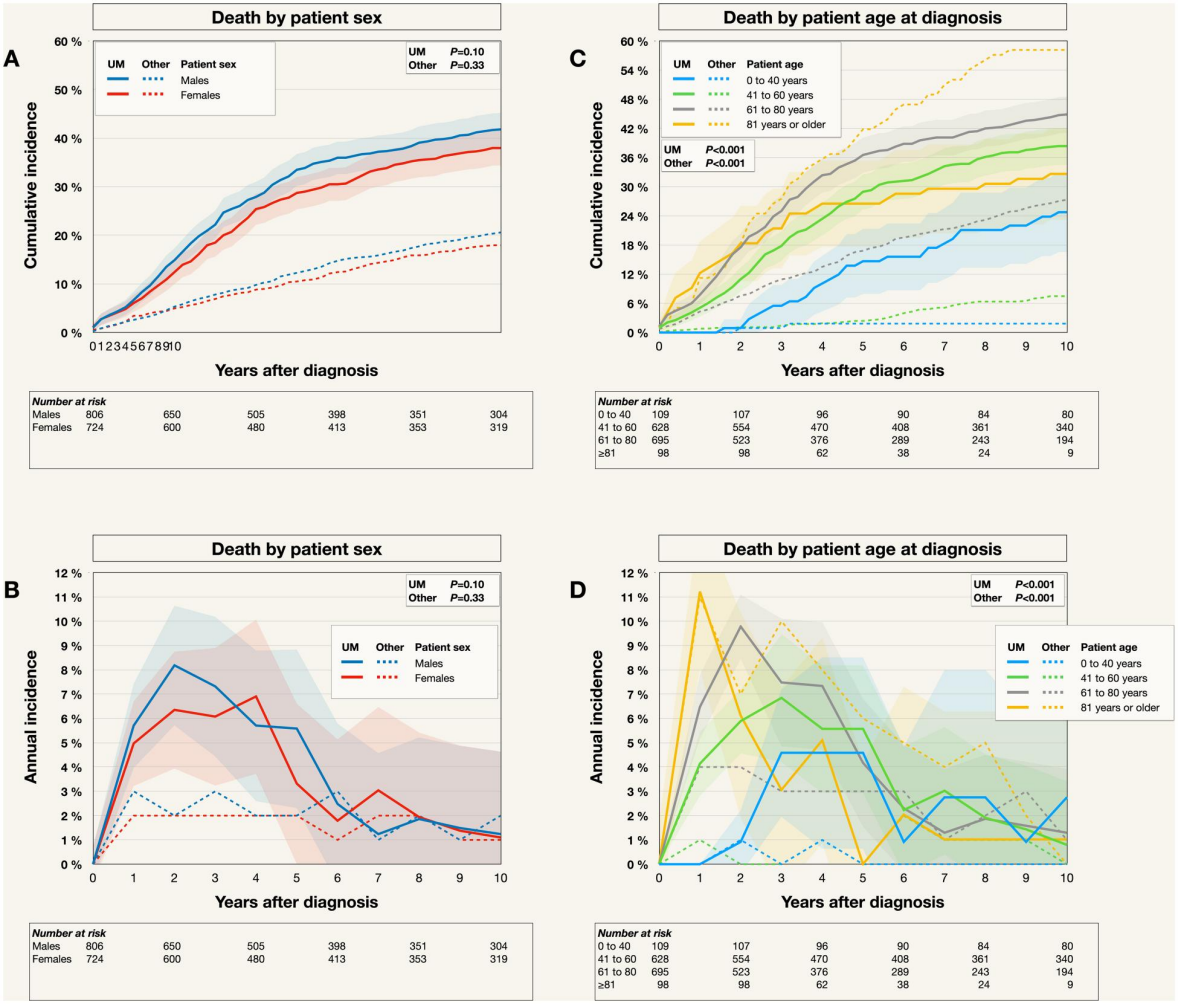
Mortality by patient sex and age in the first decade after diagnosis. **A** and **B**) The differences in mortality from metastatic uveal melanoma (UM mortality, **solid lines**) and any other cause (**dashed lines**) were not statistically significant on the .05 level. **C** and **D**) UM mortality and mortality from other causes were, however, statistically significantly different between patients who were 40 or younger; 41 to 60; 61 to 80; or at least 81 years at the time of diagnosis, with the oldest patients initially suffering from the highest UM mortality and mortality from other causes. Colored fields indicate 95% confidence intervals.

When examining the first decade after diagnosis more closely, the earlier peak UM mortality for male and older patients could be discerned more clearly (Figure 6).

## Discussion

This study provides a comprehensive overview of the causes of death within a consecutively diagnosed cohort of uveal melanoma patients. In our competing risk analyses, we observed that 48% of patients ultimately succumbed to metastatic uveal melanoma, making it the leading cause of death in cumulative and annual terms during the first 41 and 5 years post-diagnosis, respectively. This adds to findings in previous studies, in which metastatic uveal melanoma has been the leading cause of death throughout shorter follow-up periods (11). Notably, instances of metastatic deaths were recorded as late as 49.6 years after the initial diagnosis. Cardiovascular diseases, other cancers, and stroke emerged as the second, third, and fourth most common causes of death, respectively.

The system of personal identification numbers, centralized care with all Swedish patients diagnosed at a single center, and nationwide registries allow for complete control over each patient’s follow-up, regardless of their location within the country. The only exceptions in the present study were the 5 patients (0.3% of 1530) who emigrated abroad and whose current whereabouts are unknown. Another advantage is the determination of incidence derived from competing risk data, in contrast to Kaplan-Meier analyses or similar methods. When competing risks are present, the latter approach can yield erroneous outcomes due to an inherent tendency to overestimate the risk of mortality from a particular cause. This is clearly illustrated herein by greatly exaggerated rates of death from metastatic uveal melanoma, cardiovascular diseases, and other causes. Interestingly, relative survival was more than 100% four decades after diagnosis. This indicates that very long-term survivors have a survival advantage to individuals of the same sex and age from the general population. We have observed this phenomenon before, and, hypothetically, the U-shaped relative survival curve may be explained by a health-seeking behavior among uveal melanoma survivors and an accumulation of other risk factors (eg, cardiovascular morbidity and smoking) in patients dying from uveal melanoma (18,19). Additionally, to the best of the author’s knowledge, no prior investigation has successfully documented deaths resulting from metastatic uveal melanoma and other distinct causes, encompassing cardiovascular ailments and other forms of cancer, across such a long period of time.

In a previous study involving a Swedish population, the estimated 40-year mortality from cardiovascular diseases among individuals who were 60 years of age at baseline was approximately 12% (20). In contrast, our analysis revealed a 40-year rate of 23%. The discrepancies between our findings and those of the earlier study may be attributed to methodological variances. Particularly noteworthy is the fact that the participants in the previous study were required to be free of diabetes and cardiovascular diseases at the time of inclusion, both of which are well established as potent risk factors for cardiovascular-related mortality. No such criteria were applied to our study.

The cumulative lifetime risk of colorectal carcinoma-related mortality in the Western general population is approximately 1% for men and 0.7% for women. Corresponding figures stand at 4% and 3% for lung cancer, 2% for prostate cancer, and 1.3% and 0.6% for stomach cancer in men and women, respectively (21-25). Consequently, this cohort of patients diagnosed with uveal melanoma exhibited a marginally lower risk of lung carcinoma-related mortality, with a 60-year mortality of 1.4% (relative risk = 0.4, absolute risk difference = 2.1%, calculated based on an average lifetime risk of 3.5% for men and women from the general population). Similarly, mortality from colorectal and stomach carcinoma, at 1.4% and 0.9%, respectively, closely paralleled the general population's figures. Moreover, given that male patients comprised half of the included cohort (resulting in a 60-year mortality from prostate cancer of 2.4% for male patients), the incidence of death from prostate carcinoma mirrored that of the general population. It is important to note that these figures exclusively account for deaths occurring subsequent to uveal melanoma diagnosis. Consequently, the actual lifetime incidences of other cancers within the present cohort are likely to be higher. Previous studies from the United States, Sweden, and Denmark have reported an excess cancer risk for uveal melanoma patients of 11% to 21% (26-28). Compared with non-cancer controls, cancer survivors undergo more screening examinations, which may affect the reported incidence.

There was no difference in UM mortality for men and women, which corroborates previous Kaplan-Meier analyses (29). The incidence tended to be higher for men in the first decade after diagnosis, which was also seen in a recently published cohort treated mainly with plaque brachytherapy in which male patients had a higher risk of UM mortality in the first decade, after which female survivors had a higher risk (14). The statistically significantly higher UM mortality seen for the oldest patients is noteworthy. This phenomenon could indicate a lead-time bias, with older patients being at risk of having more advanced disease at the time of diagnosis. Nevertheless, ocular oncologists should be aware of the importance of patient age and its utility in prognostication (30).

This study has several limitations. First, being focused on causes of death, no data were collected regarding the anatomic extent, histological attributes, or genetic characteristics of the included primary tumors. Access to such information would have facilitated additional correlations between these factors and the causes of death. Second, despite the comprehensive data available on underlying causes of death and causative diagnoses, categorizing the cause of death presents a notable challenge, especially when multiple severe conditions coexist. Furthermore, certain conditions may exhibit similarities and overlap. For instance, deaths attributed to heart failure might alternatively be classified as deaths resulting from chronic ischemic heart disease, and vice versa. To mitigate such complexities, causes of death were aggregated into 20 distinct groups (eg, cardiovascular diseases) for the majority of analyses. The similarity between UM mortality (31%, 40%, and 45% at 5, 10, and 20 years, respectively) and the complementary values of 1 minus relative survival rates (35%, 50%, and 43% at 5, 10, and 20 years, respectively) during the initial 2 decades after diagnosis, when the majority of uveal melanoma-related fatalities occurred, suggests an acceptable accuracy in the classification of the cause of death. Third, all patients were treated with enucleation, and other treatment modalities such as plaque brachytherapy were not represented. However, it is well established that the choice between these primary tumor treatments has no effect on mortality (9,31).

Last, it is important to acknowledge that uveal melanoma is characterized by a relative lack of effective treatment regimens for stage IV disease. In contrast to cutaneous melanoma, checkpoint inhibitors rarely confer durable remissions in patients with metastatic uveal melanoma (32). Despite this, there have been some improvements since 1979 that will not be reflected in this study. In several countries, there has been a slight reduction in the average tumor size at the time of diagnosis, contributing to a modestly improved prognosis (15,33). Encouragingly, there are also emerging developments in the field: the bi-specific T-cell engager tebentafusp and isolated hepatic perfusion with melphalan are displaying promising potential for metastatic disease (1,34).

## Conclusions

This detailed analysis of 1530 Swedish patients offers valuable insights into mortality patterns and trends after a primary uveal melanoma diagnosis, revealing a notable interplay between metastatic uveal melanoma and other primary causes of death. A peak in the annual UM mortality during the second and third year post-diagnosis, and a gradual decline thereafter, was identified. Remarkably, the longest interval between uveal melanoma diagnosis and metastatic death was 49.6 years, implying that patients cannot be considered cured until achieving about 50 metastasis-free years post-diagnosis. Hence, these results highlight the crucial necessity for prolonged monitoring and deliberate interventions, particularly among high-risk groups. Age-related variations in death from metastatic uveal melanoma and other causes have been identified, demonstrating that older patients suffer from higher mortality rates in the initial years after a melanoma diagnosis. The noted variations in mortality rates also offer crucial insights that can help adapt health care strategies and support infrastructures for patients and their families.

In essence, these findings not only enrich our understanding of the disease trajectory but also carry important implications for patient and relative information, resource allocation in managing uveal melanoma and its concomitant complications, and future clinical trial designs.

## Supplementary Material

pkad097_Supplementary_Data

## Data Availability

Patient-level data on the underlying causes of death are available on Figshare: 10.6084/m9.figshare.23944203. Data are anonymized, causes of death are grouped into the 20 categories described in Table 2, patient ages at the time of uveal melanoma diagnosis and death are rounded to the nearest decade, and follow-up times are rounded to the nearest year to protect privacy.
