# Supplementary material for: Comprehensive causes of death in uveal melanoma: mortality in 1530 consecutively diagnosed patients followed until death
Source: JNCI Cancer Spectr. 2023 Nov 16;7(6):pkad097. doi: 10.1093/jncics/pkad097 (PMC10724524; doi:10.1093/jncics/pkad097)
Supplement: pkad097_Supplementary_Data [file pkad097_supplementary_data.pdf]

Supplementary Material to

Comprehensive Causes of Death in Uveal Melanoma: Mortality in 1530 Consecutive Patients Followed from Diagnosis to Death

Gustav Stålhammar, M.D. Ph.D.[1,2]

[1]St. Erik Eye Hospital, Stockholm, Sweden

[2]Department of Clinical Neuroscience, Division of Ophthalmology and Vision, Unit of Ocular Oncology and Pathology, Karolinska Institutet, Stockholm, Sweden

CONTENTS:

CAUSE OF DEATH CERTIFICATES 2

AUDIT 2

SUPPLEMENTARY TABLE 1 4

SUPPLEMENTARY TABLE 2 7

SUPPLEMENTARY FIGURE 1 13

SUPPLEMENTARY FIGURE 2 14

REFERENCES 15

Cause of death certificates

Each patient's cause of death was obtained from the Swedish National Cause of Death Register, which is based on medical death certificates. Completion of these records are mandated by law and must be submitted to the National Board of Health and Welfare within 3 weeks of death. The certificate is typically completed by a family physician, the physician last seeing the patient before death, or a pathologist.[1] By tradition, autopsy rates have been high in the Swedish population. In the 1980s, >80% of all deceased inhabitants underwent autopsy. In the 1990s, this fell to 34%, followed by even lower rates in recent years.[2] For patients dying from a diagnosed or suspected cancer, autopsy rates have remained relatively high, especially when the cause of death cannot be determined by other means, e.g., radiological examinations, biopsies or laboratory tests before death.[3] The cause of death certificate includes information about the terminal cause of death, and what diagnoses it was a consequence of. E.g., cardiac arrest can be a consequence of acute myocardial infarction, which in turn can be a consequence of atherosclerotic heart disease. Up to 15 diagnoses that lead to the terminal cause of death can be specified on the certificate. For all patients, an underlying cause of death is also determined. This is the final judgement on the fundamental reason for a death. E.g., if a patient with extensive metastatic disease in the brain, lungs, liver, and myocardium develops organ failure and dies from circulatory insufficiency, the underlying cause of death is metastatic uveal melanoma and not heart disease. All diagnoses were coded according to ICD 7 between 1960 and 1965, ICD 8 between 1966 and 1986, ICD 9 between 1987 and 1996, and ICD 10 from 1997.

Audit

Each registered underlying causes of death and causative diagnoses were audited by the author. Two hundred and forty-two different underlying causes of death had been registered for the 1530 included patients in the National Cause of Death Register. Some of these causes of death overlapped to due dissimilar terminology for the same pathology between ICD version 7 to 10. E.g., in ICD 9, code 414.0: "Coronary atherosclerosis" would equate code I25.1: "Atherosclerotic heart disease" in ICD 10. When accounting for this overlap of terms, there were 187 underlying causes of death in the data (Supplementary Table 1). In turn, these 187 underlying causes of death were a consequence of a total of 623 named diagnoses. Each patient's underlying cause of death had one to 15 such causative diagnoses registered. Again, there were some overlap between ICD versions, and

the true number of causative diagnoses was lower. Terminology from the World Health Organization (WHO) was used for the simplified terms.[4]

Further, misclassifications in the cause of death registry are not uncommon. Previous research indicates that death from metastatic uveal melanoma is often recorded as death from metastatic cutaneous melanoma.[5] Neither ICD 8 nor 9 include specific terms for uveal melanoma and its metastases. In ICD 8, the most appropriate term would be 190: “malignant neoplasm of eye”, and in ICD 9 190.6: “malignant neoplasm of the choroid”. If either term was used for a patient’s underlying cause of death, the audited cause of death was classified as metastatic uveal melanoma. If a patient diagnosed with uveal melanoma was recorded has having died from metastatic cutaneous melanoma, but had no record of neither primary nor metastatic cutaneous melanoma, the underlying cause of death was considered to be metastatic uveal melanoma.

Supplementary Table 1. Number of patients diagnosed and dead per calendar year

Year	Diagnosed, *n* (%)	Dead, *n* (%)
1960	75 (5)	5 (<1)
1961	76 (5)	6 (<1)
1962	92 (6)	17 (1)
1963	77 (5)	23 (2)
1964	77 (5)	39 (3)
1965	78 (5)	41 (3)
1966	78 (5)	39 (3)
1967	80 (5)	36 (2)
1968	87 (6)	45 (3)
1969	87 (6)	36 (2)
1970	83 (5)	54 (4)
1971	70 (5)	37 (2)
1972	75 (5)	58 (4)
1973	76 (5)	64 (4)
1974	68 (4)	50 (3)
1975	81 (5)	51 (3)
1976	79 (5)	63 (4)
1977	77 (5)	45 (3)
1978	72 (5)	81 (5)
1979	42 (3)	58 (4)
1980	0 (0)	53 (4)
1981	0 (0)	43 (3)
1982	0 (0)	46 (3)
1983	0 (0)	42 (3)
1984	0 (0)	35 (2)
1985	0 (0)	21 (1)
1986	0 (0)	33 (2)
1987	0 (0)	36 (2)
1988	0 (0)	26 (2)

1989	0 (0)	18 (1)
1990	0 (0)	18 (1)
1991	0 (0)	18 (1)
1992	0 (0)	12 (1)
1993	0 (0)	11 (1)
1994	0 (0)	13 (1)
1995	0 (0)	17 (1)
1996	0 (0)	16 (1)
1997	0 (0)	16 (1)
1998	0 (0)	11 (1)
1999	0 (0)	18 (1)
2000	0 (0)	11 (1)
2001	0 (0)	11 (1)
2002	0 (0)	12 (1)
2003	0 (0)	11 (1)
2004	0 (0)	8 (1)
2005	0 (0)	4 (<1)
2006	0 (0)	10 (1)
2007	0 (0)	10 (1)
2008	0 (0)	10 (1)
2009	0 (0)	13 (1)
2010	0 (0)	9 (1)
2011	0 (0)	5 (<1)
2012	0 (0)	10 (1)
2013	0 (0)	1 (<1)
2014	0 (0)	3 (<1)
2015	0 (0)	6 (<1)
2016	0 (0)	7 (1)
2017	0 (0)	4 (<1)
2018	0 (0)	9 (1)
2019	0 (0)	4 (<1)
2020	0 (0)	9 (1)
2021	0 (0)	3 (<1)

2022	0 (0)	8 (1)
2023	0 (0)	1 (<1)

**Supplementary Table 2**. Registered underlying cause of death for all 1530 patients.			
	**Cause of death**	***n***	**%**
1	Cutaneous melanoma	256	16.7
2	Malignant neoplasm of eye	223	14.6
3	Metastatic uveal melanoma	186	12.2
4	Acute myocardial infarction	128	8.4
5	Chronic ischemic heart disease	103	6.7
6	Cerebrovascular disease	40	2.6
7	Malignant neoplasm without specification of site	23	1.5
8	Lung cancer	20	1.3
9	Pneumonia	20	1.3
10	Prostate cancer	20	1.3
11	Atherosclerotic heart disease	21	1.4
12	Heart failure	18	1.2
13	Acute pericarditis	17	1.1
14	Cerebral haemorrhage	15	1.0
15	Stomach cancer	15	1.0
16	Secondary malignant neoplasm of respiratory and digestive system	14	0.9
17	Cerebral thrombosis	13	0.8
18	Malignant neoplasm of large intestine, except rectum	13	0.8
19	Atherosclerosis NOS	12	0.8
20	Dementia	11	0.7
21	Malignant neoplasm of liver and intrahepatic bile ducts, specified as primary	11	0.7
22	Pulmonary embolism and infarction	11	0.7
23	Accidental fall	10	0.7
24	Alzheimer disease	10	0.7
25	Diabetes mellitus	10	0.7
26	Breast cancer	9	0.6
27	Heart disease, unspecified	9	0.6
28	Pancreatic cancer	9	0.6

29	Senility without mention of psychosis	9	0.6
30	Acute myocarditis	8	0.5
31	Cerebral infarction	8	0.5
32	Malignant neoplasm of brain	8	0.5
33	Cirrhosis of liver	5	0.3
34	Malignant neoplasm of other and unspecified urinary organs	5	0.3
35	Patient emigrated (i.e., cause of death not available)	5	0.3
36	Tuberculosis	5	0.3
37	Bronchitis	4	0.3
38	Chronic obstructive pulmonary disease	4	0.3
39	Colorectal cancer	4	0.3
40	Generalized and unspecified atherosclerosis	4	0.3
41	Infections of kidney	4	0.3
42	Malignant neoplasm of ovary, fallopian tube or broad ligament	4	0.3
43	Malignant neoplasm of rectum and rectosigmoid junction	4	0.3
44	Sepsis	4	0.3
45	Subarachnoid haemorrhage	4	0.3
46	Alcoholism	3	0.2
47	Aortic aneurysm	3	0.2
48	Cholecystitis and cholangitis, without mention of calculi	3	0.2
49	Chronic disease of aortic valve	3	0.2
50	Diseases of mitral valve	3	0.2
51	Malignant neoplasm of bladder	3	0.2
52	Multiple myeloma	3	0.2
53	Ruptured aortic aneurysm	3	0.2
54	Secondary malignant neoplasm of respiratory and digestive systems	3	0.2
55	Sequelae of cerebrovascular disease	3	0.2
56	Suicide	3	0.2
57	Traumatic amputation of arm and hand (complete) (partial)	3	0.2
58	Acute kidney failure	2	0.1
59	Anemia	2	0.1
60	Aortic (valve) stenosis	2	0.1

61	Asthma	2	0.1
62	Atherosclerotic cardiovascular disease	2	0.1
63	Atrial fibrillation and flutter	2	0.1
64	Chronic bronchitis	2	0.1
65	Dissection of aorta	2	0.1
66	Fracture of neck of femur	2	0.1
67	Gangrene	2	0.1
68	Gastrointestinal haemorrhage	2	0.1
69	Left ventricular failure	2	0.1
70	Malignant neoplasm of gallbladder and bile ducts	2	0.1
71	Malignant neoplasm of liver and intrahepatic bile ducts9	2	0.1
72	Motor vehicle traffic accident	2	0.1
73	Multiple and unspecified open wound of upper limb	2	0.1
74	Non-melanoma skin cancer	2	0.1
75	Other diseases of intestines and peritoneum	2	0.1
76	Other ill-defined and unspecified causes of mortality	2	0.1
77	Other myocardial insufficiency	2	0.1
78	Other peripheral vascular disease	2	0.1
79	Other venous embolism and thrombosis	2	0.1
80	Parkinson disease	2	0.1
81	Pulmonary edema	2	0.1
82	Renal failure	2	0.1
83	Systolic heart failure	2	0.1
84	Ulcer of stomach	2	0.1
85	Ventricular fibrillation and flutter	2	0.1
86	Vitamin A deficiency	2	0.1
87	Arterial embolism and thrombosis	1	0.1
88	Malignant neoplasm of thyroid gland	1	0.1
89	Pulmonary embolism and infarction	1	0.1
90	Acute and subacute endocarditis	1	0.1
91	Acute bronchitis	1	0.1
92	Acute bronchitis and bronchiolitis	1	0.1
93	Acute Myeloid leukemia	1	0.1

94	Acute vascular disorders of intestine	1	0.1
95	Affective psychoses	1	0.1
96	Alcohol dependence syndrome	1	0.1
97	Arteriosclerosis	1	0.1
98	Arteriosclerosis of aorta	1	0.1
99	Ben neoplasm cerebr meninges	1	0.1
100	Benign neoplasm of brain and other parts of nervous system	1	0.1
101	Benign neoplasm of endocrine glands	1	0.1
102	Cardiac arrest, cause unspecified	1	0.1
103	Cardiomyopathy, unspecified	1	0.1
104	Chorionepithelioma	1	0.1
105	Chronic atrial fibrillation	1	0.1
106	Chronic disease of endocardium	1	0.1
107	Chronic Lymphoid leukemia	1	0.1
108	Congestive heart failure	1	0.1
109	Cystic kidney disease	1	0.1
110	Dermatophytosis	1	0.1
111	Diabetes with other coma	1	0.1
112	Diffuse diseases of connective tissue	1	0.1
113	Elevated erythrocyte sedimentation rate	1	0.1
114	Emphysema	1	0.1
115	Endocarditis	1	0.1
116	Enteritis	1	0.1
117	Enterocolitis due to Clostridium difficile	1	0.1
118	Essential (primary) hypertension	1	0.1
119	Gastritis and duodenitis	1	0.1
120	Herpes zoster	1	0.1
121	Hodgkin's disease	1	0.1
122	Hyperplasia of prostate	1	0.1
123	Hypertensive heart and renal disease with both (congestive) heart failure and renal failure	1	0.1
124	Hypertensive heart disease with (congestive) heart failure	1	0.1
125	Ileus	1	0.1

126	Ill-defined heart disease	1	0.1
127	Influenza	1	0.1
128	Intracardiac thrombosis, not elsewhere classified	1	0.1
129	Lymphatic leukaemia	1	0.1
130	Lymphoma	1	0.1
131	Lymphosarcoma	1	0.1
132	Macroglobulinemia	1	0.1
133	Mal neoplasm small bowel NOS	1	0.1
134	Malig neoplasm abdomen	1	0.1
135	Malig neoplasm gallbladder	1	0.1
136	Malig neoplasm skin NOS	1	0.1
137	Malign neoplasm adrenal	1	0.1
138	Malignant neoplasm cecum	1	0.1
139	Malignant neoplasm colon NOS	1	0.1
140	Malignant neoplasm of bones of skull and face	1	0.1
141	Malignant neoplasm of Bones of skull and face	1	0.1
142	Malignant neoplasm of cervix uteri	1	0.1
143	Malignant neoplasm of connective and soft tissue	1	0.1
144	Malignant neoplasm of heart	1	0.1
145	Malignant neoplasm of Intestinal tract, part unspecified	1	0.1
146	Malignant neoplasm of kidney, except renal pelvis	1	0.1
147	Malignant neoplasm of liver and intrahepatic bile ducts	1	0.1
148	Malignant neoplasm of nasopharynx	1	0.1
149	Malignant neoplasm of oesophagus	1	0.1
150	Malignant neoplasm of other and unspecified parts of the Buccal mucosa	1	0.1
151	Malignant neoplasm of other part of nervous system	1	0.1
152	Malignant neoplasm of peritoneum and retroperitoneal tissue	1	0.1
153	Malignant neoplasm of pleura	1	0.1
154	Malignant neoplasm of rectum	1	0.1
155	Malignant neoplasm of small intestine, including duodenum	1	0.1
156	Malignant neoplasm of trachea, bronchus and lung	1	0.1
157	Malignant neoplasm of uterus	1	0.1

158	Malignant neoplasm tonsil	1	0.1
159	Motor neuron disease	1	0.1
160	Motor vehicle traffic accident involving collision with another motor vehicle	1	0.1
161	Myeloid leukaemia	1	0.1
162	neoplasm of uncertain or unknown behaviour of colon	1	0.1
163	neoplasm of unspecified nature of eye, brain and other parts of nervous system	1	0.1
164	neoplasm of unspecified nature of other female genital organs	1	0.1
165	Non-Hodgkin lymphoma	1	0.1
166	Old myocardial infarction	1	0.1
167	Open wound of eye and orbit	1	0.1
168	Other and unspecified noninfectious gastroenteritis and colitis	1	0.1
169	Other deafness	1	0.1
170	Other hernia of abdominal cavity with obstruction	1	0.1
171	Other neoplasms of lymphoid tissue	1	0.1
172	Other renal disease	1	0.1
173	Pancarditis	1	0.1
174	Peripheral vascular disease, unspecified	1	0.1
175	Presenile dementia	1	0.1
176	Renal sclerosis	1	0.1
177	Senile and presenile dementia	1	0.1
178	Sequelae of cerebrovascular disease8	1	0.1
179	Superficial injury of trunk	1	0.1
180	Suppurative and unspecified otitis media	1	0.1
181	Thyrotoxicosis	1	0.1
182	Traumatic spinal cord injury	1	0.1
183	Unspecified hypertensive heart disease	1	0.1
184	Unspecified hypertensive kidney disease	1	0.1
185	Viral pneumonia	1	0.1
186	Volvulus	1	0.1
	Sum	1530	100.0

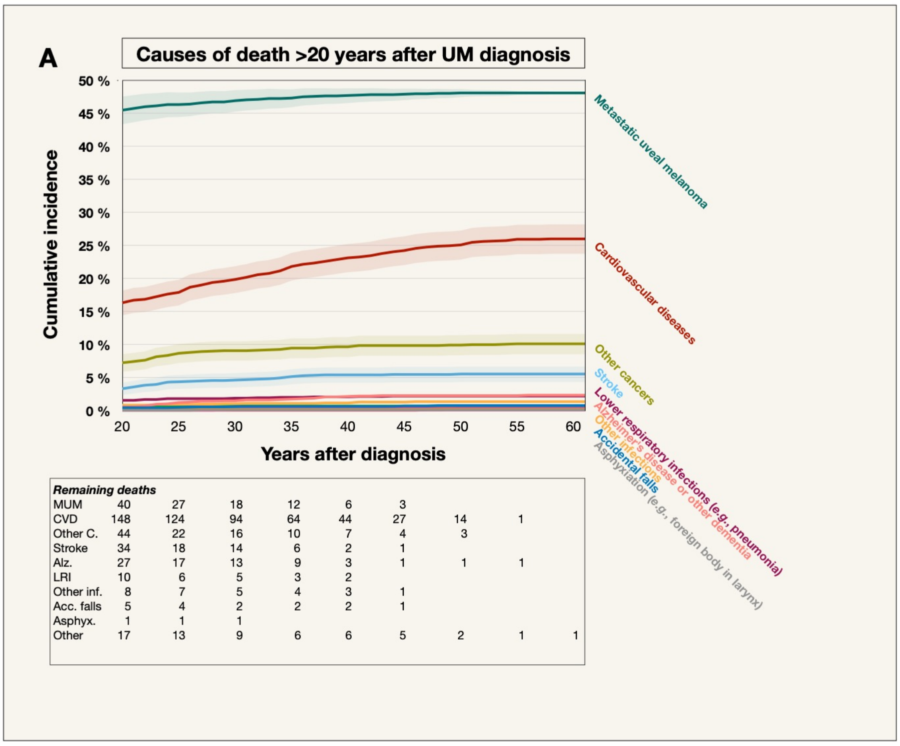

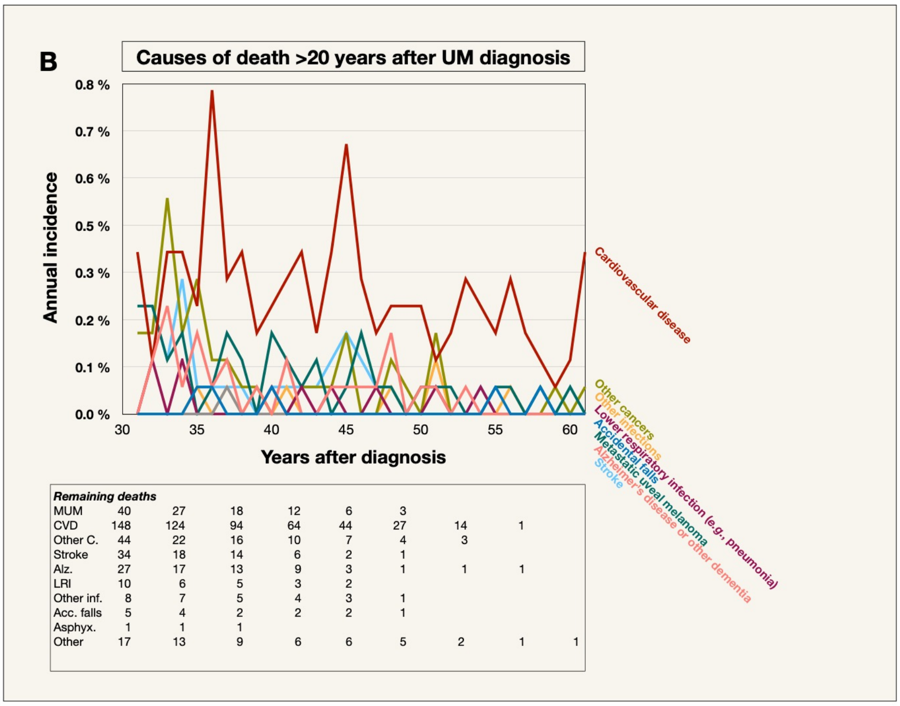

Supplementary figure 1. Causes of death >20 years after uveal melanoma (UM) diagnosis. A) Cumulative incidence of death from metastatic uveal melanoma (MUM); cardiovascular diseases (CVD); Other cancers (Other c.); Stroke; Alzhemier's disease or other dementia (Alz.); Lower respiratory infections (LRI); Other infections (Other inf.); Accidental falls (Acc. falls.); Asphyxiation (Asphyx.); and other causes (Other). B) Annual incidence. Colored fields in A represent 95% confidence intervals.

Supplementary figure 2.

Kaplan-Meier curves for the four most common causes of death (A to D), and all other causes combined (E). Colored fields indicate 95% confidence intervals. Strata = all 1530 patients.

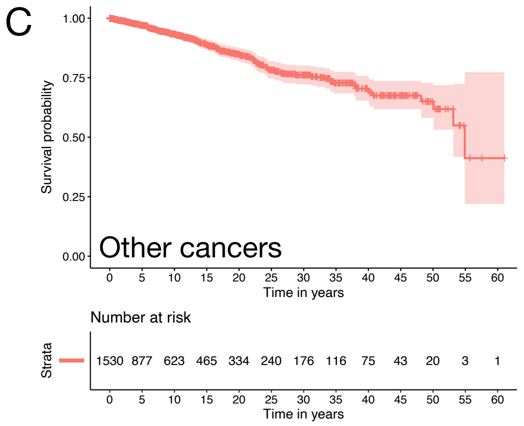

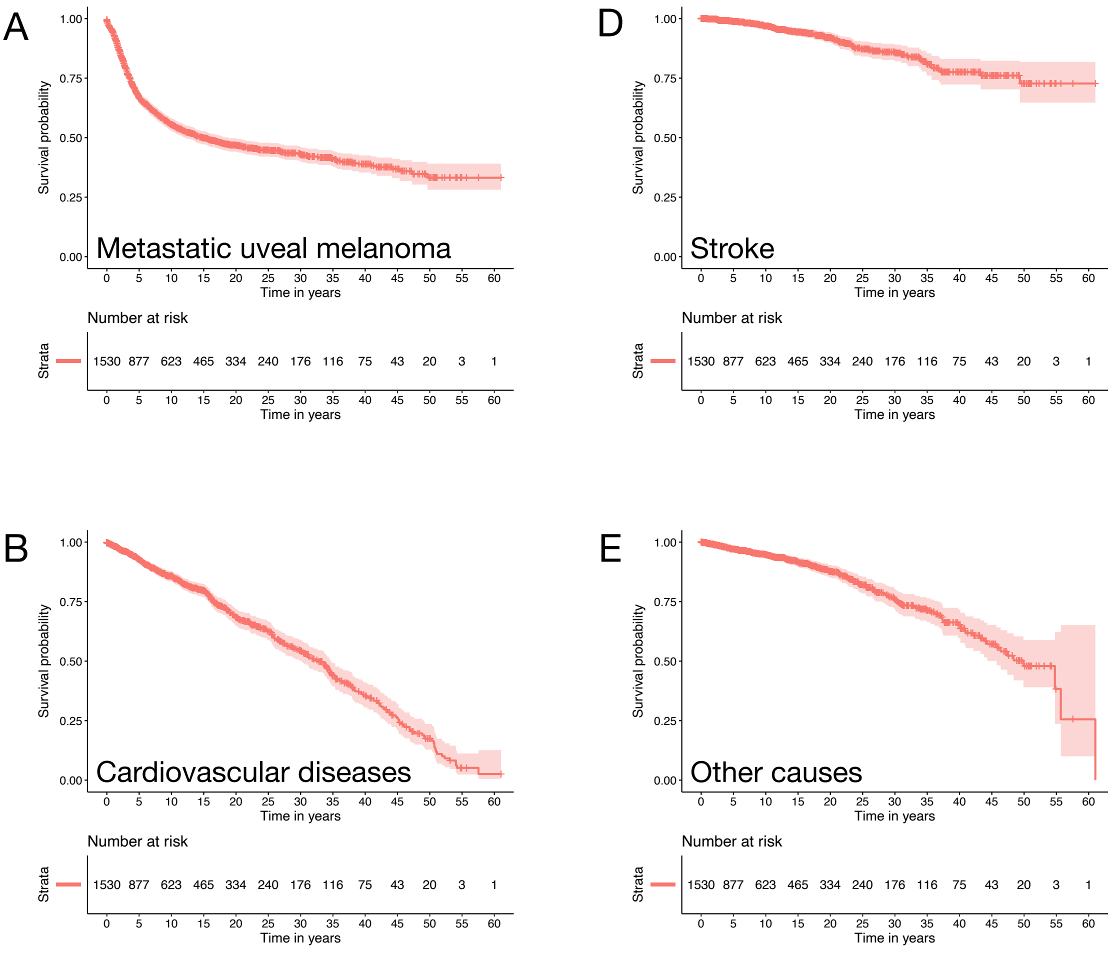

References

1. Brooke H, Talbäck M, Hörnblad J, *et al.* The Swedish cause of death register. European Journal of Epidemiology 2017;32(9):765-773.
2. Lindstrom P, Janzon L, Sternby NH. Declining autopsy rate in Sweden: a study of causes and consequences in Malmo, Sweden. J Intern Med 1997;242(2):157-65.
3. Nystrom L, Larsson LG, Rutqvist LE, *et al.* Determination of cause of death among breast cancer cases in the Swedish randomized mammography screening trials. A comparison between official statistics and validation by an endpoint committee. Acta Oncol 1995;34(2):145-52.
4. Foreman KJ, Marquez N, Dolgert A, *et al.* Forecasting life expectancy, years of life lost, and all-cause and cause-specific mortality for 250 causes of death: reference and alternative scenarios for 2016–40 for 195 countries and territories. The Lancet 2018;392(10159):2052-2090.
5. Bergman L, Seregard S, Nilsson B, *et al.* Uveal melanoma survival in Sweden from 1960 to 1998. Investigative ophthalmology & visual science 2003;44(8):3282.
